# Failure to Identify the Left Arcuate Fasciculus at Diffusion Tractography Is a Specific Marker of Language Dysfunction in Pediatric Patients with Polymicrogyria

**DOI:** 10.1155/2015/351391

**Published:** 2015-05-28

**Authors:** Michael J. Paldino, Kara Hedges, Nadine Gaab, Albert M. Galaburda, P. Ellen Grant

**Affiliations:** ^1^Department of Radiology, Boston Children's Hospital and Harvard Medical School, Boston, MA 02115, USA; ^2^Developmental Medicine Center, Boston Children's Hospital and Harvard Medical School, Boston, MA 02115, USA; ^3^Department of Neurology, Beth Israel Deaconess Medical Center and Harvard Medical School, Boston, MA 02215, USA; ^4^Departments of Radiology and Newborn Medicine, Boston Children's Hospital and Harvard Medical School, Boston, MA 02115, USA

## Abstract

*Background*. Polymicrogyric cortex demonstrates interindividual variation with regard to both extent of dyslamination and functional capacity. Given the relationship between laminar structure and white matter fibers, we sought to define the relationship between polymicrogyria (PMG), intrahemispheric association pathways, and network function. *Methods*. Each arcuate fasciculus (AF) was categorized as present or absent. Language was characterized by a pediatric neurologist. The presence of dysplastic cortex in the expected anatomic locations of Broca's (BA) and Wernicke's areas (WA) was evaluated by two pediatric neuroradiologists blinded to DTI and language data. *Results*. 16 PMG patients and 16 age/gender-matched controls were included. All normative controls had an identifiable left AF. 6/7 PMG patients with dysplastic cortex within BA and/or WA had no left AF; PMG patients without involvement of these regions had a lower frequency of absence of the left AF (*p* < 0.006). All patients without a left AF had some degree of language impairment. PMG patients without a left AF had a significantly greater frequency of language impairment compared to those PMG patients with a left AF (*p* < 0.003). *Conclusion*. In patients with PMG (1) the presence of dysplastic cortex within WA and/or BA is associated with absence of the left AF and (2) absence of the left AF is associated with language impairment.

## 1. Introduction

Polymicrogyria (PMG) is a malformation of cortical development that arises as a result of disturbed cortical organization and late neuronal migration [1]. It is characterized macroscopically by an excessive number of small gyri and microscopically by abnormal lamination of the involved cortex [2]. PMG demonstrates substantial inter- and intrapatient pathologic heterogeneity [3]. Similarly, the functional capacity of polymicrogyric cortex is highly variable, ranging from absent to pathogenic (e.g., epilepsy) to normal [4, 5]. Functional MRI has been used to evaluate the functional capacity of polymicrogyric cortex [4]. However, the identification of a task-induced blood oxygen level dependent (BOLD) effect within a given region of cortex does not necessarily imply normal function. Furthermore, it is not clear to what degree this approach is sensitive to clinically relevant residual, though substantially reduced, function [6]. Considering that a subset of patients with PMG patients are candidates for epilepsy surgery, information regarding the functional status of malformed cortex would be invaluable to presurgical planning.

The normal human cerebral neocortex is composed of six discrete layers. In very general terms, layer 1 contains few neurons which primarily receive interhemispheric fibers. Pyramidal neurons in layers 2 and 3 originate and receive intra- and interhemispheric corticocortical fibers. Layer 4 is the principal target of thalamocortical fibers. Layer 5 is the major source of projection fibers to the basal ganglia, brainstem, and spinal cord. Finally, fibers originating in layer 6 primarily project to the thalamus. Although throughout the neocortex this fundamental organization is essentially invariant, the relative contribution of each layer to total cortical thickness reflects, to a significant degree, functional specialization [7]. For example, association cortices tend to have relatively well-developed layer 3, an important source of corticocortical fibers. Layer 5, on the other hand, the major source of subcortical projection pathways, is particularly prominent in the primary motor cortex. This relationship between white matter connections and laminar structure suggests that the nature of visualized connections might be related to the structure of the cortex itself.

Polymicrogyric cortex contains essentially the same principal classes of neurons as normal cortex; furthermore, neuronal subtypes are located at appropriate, or at least analogous, laminar levels [8–10]. Pathologic and experimental studies have consistently demonstrated substantial damage to large pyramidal cells in layer 5, leaving a cell sparse zone that is nearly devoid of neuronal elements (the lamina dissecans) [9, 11, 12]. These same studies have demonstrated relative, though variable, preservation of superficial cortical layers 2 and 3 [8, 13]. Recent work has raised the possibility that this variation in the integrity of superficial layers could account in part for the breadth of functional capacity retained by polymicrogyric cortex [6]. As the above observations predict a consistent and severe impact on subcortical projection pathways with variable sparing of corticocortical association pathways, we hypothesize that interrogation of association pathways may provide a noninvasive assessment of the structure and function of polymicrogyric cortex in an individual patient.

The arcuate fasciculus (AF) is a major intrahemispheric association pathway which connects two cortical areas that play a major role in human language, Wernicke's (WA) and Broca's areas (BA) [14, 15]. Although the exact role(s) of the AF remains the subject of debate, there is strong evidence to suggest an important contribution to language function [16, 17]. In contradistinction to the right AF whose presence is highly variable, the left AF has been consistently identified in normal subjects [18, 19]. Furthermore, patients with developmental delay, which is virtually always associated with significant language delay, seem to demonstrate abnormalities of the left AF at a higher frequency than normal subjects [18–21]. Together, these data suggest that formation of the left AF may be an important step toward the development of normal language function. Although patients with PMG have a high prevalence of language difficulties, there are few data regarding the relationship between language dysfunction and the status of the AF in this patient population [22]. Hence, the goal of this study was to define the relationship between polymicrogyric cortex, abnormalities within associated intrahemispheric association pathways, and network function. In particular, we sought to test the following two hypotheses in pediatric patients with PMG:

1. There is an association between the presence of dysplastic cortex within the expected anatomic locations of WA and/or BA and absence of the left AF at diffusion tractography.
2. There is an association between absence of the left AF and language impairment.

## 2. Methods

This HIPAA-compliant study was approved by the local institutional review board. Patients were identified retrospectively with the following inclusion criteria: (1) pediatric age group (less than or equal to 18 years); (2) diagnosis of polymicrogyria (PMG) established by MRI; (3) MRI of the brain performed at 3 Tesla, including DTI; (4) language development characterized by a pediatric neurologist. Refinements to the above-defined population were based on the following exclusion criteria: (1) motion or other degradations to image quality; (2) exclusion of patients younger than 3 years of age, to increase confidence in the clinical determination of language delay.

An age- and gender-matched normative control group was also retrospectively identified with the following inclusion criteria: (1) clinical indication of headache; (2) MRI of the brain performed at 3 Tesla, including DTI (identical to DTI performed in the patient group); (3) language characterized as normal by a pediatric neurologist. Exclusion criteria were (1) any neurologic abnormality by history or physical exam; (2) any degree of language impairment; (3) any MRI abnormality; (4) significant motion or other degradations to image quality. It should be noted that the normative cohort was included to further inform interpretation of results in the polymicrogyric population not to definitively define the normal character of the arcuate fasciculus. While patients with headaches may in fact demonstrate white matter differences compared to the truly normal population, the typical headache patient in our practice very commonly demonstrates no anatomic or neurologic abnormalities.

### 2.1. MR Imaging

All imaging was performed on two 3-Tesla magnets (Siemens, Tim Trio, Erlangen, Germany). The following sequences were obtained: (1) sagittal magnetization prepared rapid gradient echo (MPRAGE; TR/TE: 2530 ms/3.39 ms; 1 acquisition; Flip: 7 degrees, inversion time: 1100 ms; FOV: 22 cm; acceleration: 2; voxel (mm): 1 × 1 × 1); (2) axial FSE T2-weighted images (TR/TE: 11,730 ms/89 ms; 2 acquisitions; Flip: 120 degrees; FOV: 22 cm; acceleration: 2; voxel (mm): 0.6 × 0.4 × 2.5); (3) axial FLAIR (TR/TE: 9,000 ms/137 ms; 1 acquisition; Flip: 150 degrees; FOV: 22 cm; voxel (mm): 0.7 × 0.7 × 4); (4) axial single-shot EPI DTI (TR/TE (ms): 7000/142; Flip: 90 degrees; 1 acquisition; voxel (mm): 2 × 2 × 2). For DTI, 35 image sets were acquired, five without diffusion weighting and thirty with noncollinear diffusion-weighting gradients (*b* value: 1000 sec/mm^2^). All images were visually inspected for artifacts, including subject motion.

### 2.2. Image Processing and Analysis

**Figure 1:**
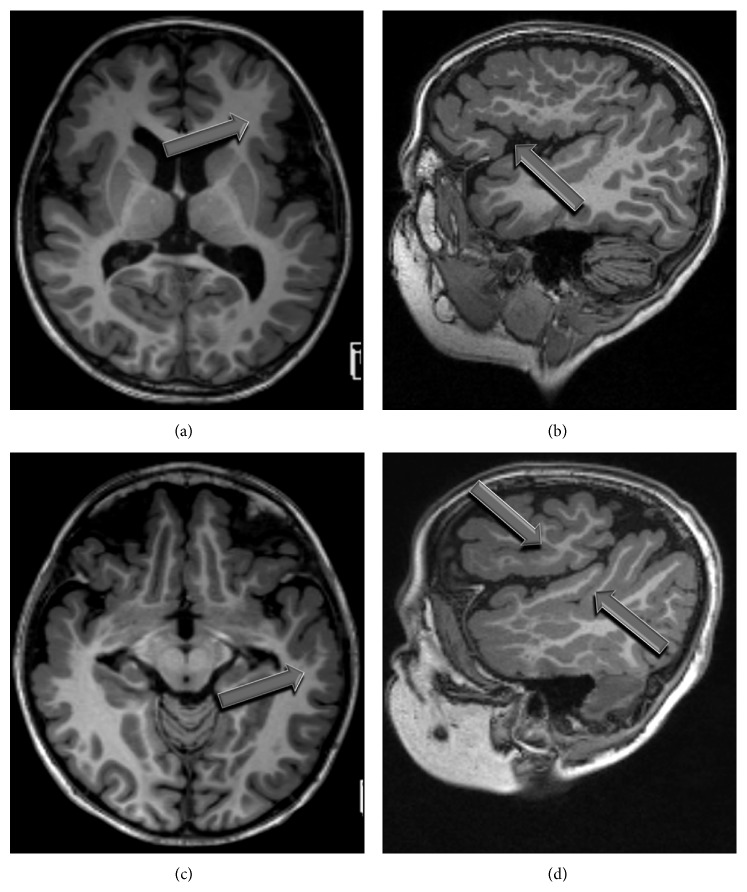
Axial (a) and sagittal (b) MPRAGE images demonstrate polymicrogyria involving the left inferior frontal gyrus. Similarly, involvement of the left posterior perisylvian region can be seen on axial (c) and sagittal (d) MPRAGE images in the same patient.

Based on structural images, the location of dysplastic cortex in PMG patients was evaluated by two pediatric neuroradiologists blinded to the DTI and language data; discordant results were settled by consensus. In particular, each patient was characterized according to the presence/absence of dysplastic cortex in the expected anatomic locations of Broca's (BA; pars opercularis and pars triangularis of the inferior frontal gyral cortex) and/or Wernicke's (WA; posterior aspect of the superior and middle temporal gyri and superior temporal sulcus) areas in the left hemisphere (see, e.g., Figure 1). Dysplastic cortex visible within any part of the area in question was considered positive for involvement of that area.

**Figure 2:**
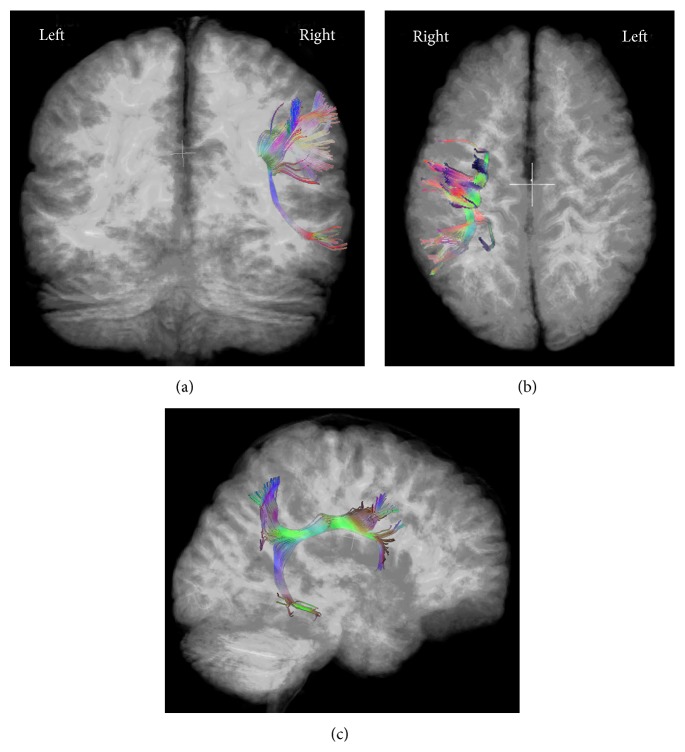
Posterior (a), superior (b), and right-lateral (c) views of a 3-dimensional tract reconstruction of the arcuate fasciculus (AF) in a patient with polymicrogyria (same patient as Figure 1). Images demonstrate presence of the AF in the right hemisphere only. This patient exhibited mild-to-moderate language impairment.

Maps of diffusivity (MD) and fractional anisotropy (FA) were created using Diffusion Toolkit (http://www.trackvis.org/). For each voxel, a tensor matrix was derived. After diagonalization of the matrix, eigenvalues were obtained and MD and FA were quantified for each pixel according to standard equations [23]. Diffusion Toolkit (http://www.trackvis.org/) was used for deterministic tract reconstruction using a fiber association by continuous tracking algorithm (35-degree angular threshold). A DWI mask was used to remove cerebrospinal fluid, a process which has been shown to effectively prevent spurious tract reconstruction [24]. Trackvis (http://www.trackvis.org/) was then used for isolation of the AF and fiber-track analysis. Regions of interest for tract segmentation were placed manually on the color FA maps cross referenced to the *b*_0_ images according to previously described methods [25]. The AF in each subject was categorized as present on the left only, on the right only, or bilaterally (Figure 2). This assessment of the AF as present versus absent has been previously shown to be highly reproducible [26]. Mean MD and mean FA were then calculated for each identifiable AF.

Patients were divided into three groups based on characterization of their language development by a pediatric neurologist: (1) intact: age-appropriate; (2) mild-to-moderate impairment: delayed by comparison to peers (either expressive or receptive); (3) profound impairment: absence of verbal language.

### 2.3. Statistics

Proportions of subjects with versus without absence of the left AF and with versus without language impairment were compared using Fisher Exact test (alpha: 0.05). The Wilcoxon rank sum test was used to compare continuous variables (mean FA and mean MD) between two groups (alpha: 0.05). The relationships between tract diffusion parameters and age were assessed using linear regression. All statistical testing was performed using SAS software, Version 9.2 (SAS Institute, Cary, NC, USA).

## 3. Results

### 3.1. Patients

Imaging was performed from January 2009 to December 2010. Of the 20 patients identified meeting the inclusion criteria, 1 was excluded on the basis of significant motion and 3 on the basis of age (less than 3 years). 16 PMG patients (age range: 3–18 years; median: 10 years; 9 males, 7 females) and 16 age- and gender-matched normative controls (age range 3–18 years; median: 10 years; 9 males, 7 females) comprised the final study group.

### 3.2. Character of the Arcuate Fasciculus

All normative controls had an identifiable left AF. Nine (56%) PMG patients had an identifiable left AF. The frequency of absence of the left AF in PMG patients was significantly higher than that in normative controls (*p* < 0.0004). There was no significant difference between either the mean FA or mean MD of the identified left AF in PMG patients versus controls. Direct and inverse linear relationships to age were observed, respectively, for left arcuate FA and MD in both the PMG patient (MD: *p* < 0.01; FA: *p* < 0.01) and normative subject (MD: *p* < 0.001; *p* < 0.004) groups. There was no significant gender difference in either the mean MD or mean FA of the identified left AF in either PMG patients or controls. Trends toward higher FA in the left versus right AF in PMG patients and controls did not meet statistical significance (PMG: *p* = 0.21; normative: *p* = 0.19).

### 3.3. Relationship between Dysplastic Cortex and the Left Arcuate Fasciculus

**Figure 3:**
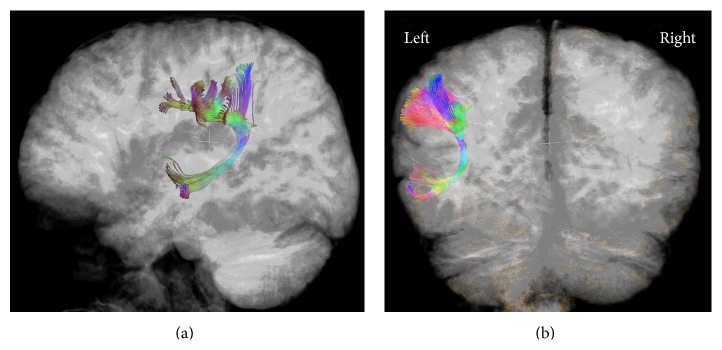
Left lateral (a) and posterior (b) views of the arcuate fasciculus (AF) in a patient with polymicrogyria involving both Broca's and Wernicke's areas in the left hemisphere. This right-handed patient had an identifiable left AF and normal language development.

**Figure 4:**
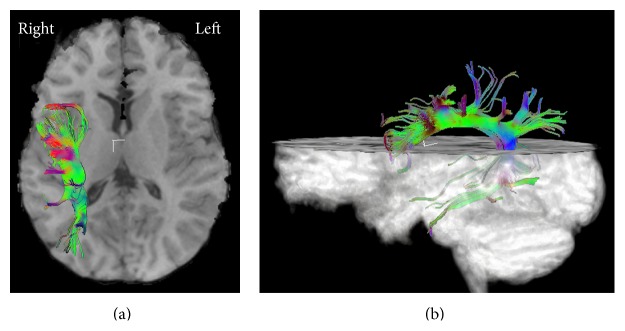
Superior (a) and left lateral (b) views of the arcuate fasciculus (AF) in a patient with no dysplastic involvement of the left hemisphere. Images demonstrate no identifiable left AF. This right-handed patient was impaired with respect to language.

Six (86%) PMG patients with dysplastic cortex within the left BA and/or WA had no left AF (see representative example, Figure 2); PMG patients with no involvement of these gyri had a significantly lower frequency of absence of the left AF (11%; *p* < 0.006). There was a single patient who had dysplastic involvement of BA and WA and an identifiable left AF (Figure 3); this patient had normal language development. Of the PMG patients with absent left AF, six (86%) had dysplastic cortex within BA and/or WA. A single patient had no identifiable left AF but no dysplastic involvement of the left BA or WA (Figure 4); this patient was impaired with respect to language.

### 3.4. Language Development

All normative subjects had intact language. By comparison, seven (44%) PMG patients had intact language. Six (38%) were mildly-to-moderately impaired and three (19%) were profoundly impaired. The extent of PMG involvement in terms of the number of lobes involved did not differ between language impaired and intact patients. The likelihood of language impairment in PMG patients was not associated with gender (*p* > 0.9).

All normative subjects had an identifiable left AF. Similarly, all PMG patients with normal language had an identifiable left AF. By contrast, all PMG patients without a left AF had some degree of language impairment; this finding was observed consistently in both right- (*n* = 4) and left- (*n* = 3) handed patients. PMG patients without a left AF had a significantly greater frequency of language impairment compared to the normative population (*p* < 0.00001). Language impairment was also significantly more prevalent in PMG patients without a left AF versus PMG patients with a left AF (*p* < 0.003). Test characteristics of absence of the left AF with respect to language impairment in an individual patient are presented in Table 1.

## 4. Discussion

Based on this study of pediatric patients with PMG, we report two main findings: (1) dysplastic cortex within the expected anatomic locations of two key language areas was associated with absence of the left AF. (2) Absence of the left AF was strongly associated with impairment of language function.

Formation of association pathways involves numerous developmental processes that act in a coordinated fashion to establish mature patterns of cerebral connectivity. These include, but are not limited to, differentiation and maturation of pyramidal cells in cortical layer 3, axonal genesis and guidance to the subplate, synapse formation and establishment of connectivity with the developing cortical plate, and selection (or pruning) of connections [27]. The last of these steps seems to involve activity-dependent stabilization of functionally useful synapses [28].

We report a significant association between the presence of dysplastic cortex within the expected anatomic locations of WA and/or BA and absence of the left AF. This observation suggests a frequent impact on the establishment and/or preservation of corticocortical connections in patients with PMG. One possible explanation for this finding is that PMG patients frequently exhibit significant alterations within the cortical layers involved in corticocortical connectivity. It would follow that abnormal cortical lamination could result in a diminished and/or abnormal contribution to the AF and, therefore, failure to detect it at tractography. It is important to note that the histopathological significance of failure to identify a tract at diffusion tractography is yet to be established. In addition to absence of the tract in an absolute sense, this finding could reflect marked disorganization of white matter structure resulting in a profound loss of tissue coherence. In other words, superficial cortical layers that are absent, significantly attenuated, or that are otherwise associated with aberrant connections between the origin and target cortices could all theoretically result in an undetectable AF. Interestingly, Phillips et al. recently demonstrated a direct relationship between the FA of the AF and cortical thickness within the two language areas connected by this pathway [29]; these findings lend credence to the notion that tract based measures of structural connectivity may indeed have value as a potential marker of cortical structure. As an alternative explanation, disordered genetic regulation of molecular events involved in the formation of corticocortical connections could also account for the reported findings. In this scenario, absence of a detectable AF need not be associated with histologic abnormality of superficial cortical layers. Although this explanation is considered less likely given the fact that many cases of PMG are not genetic in etiology, future studies with pathologic correlation will be of great interest toward definitively defining the potential for tract based measures to accurately reflect cortical structure. As a final possibility, research in epilepsy has demonstrated the presence of relatively widespread abnormal and epileptogenic networks in patients with focal structural lesions and, furthermore, has suggested the potential for ongoing seizure activity to establish and/or potentiate these networks [30–32]. Aberrant connectivity resulting from such activity-dependent reorganization could also account, at least in part, for the findings in this study.

Although it has not been well studied to date, the idea that corticocortical connections may be abnormal in PMG is consistent with a case report by Munakata et al., in which a case of unilateral left-sided PMG was associated with absence of the left AF [6]. Similarly, Bernal et al. reported absence of the AF in two cases of bilateral perisylvian polymicrogyria [22]. Further indirect support for this idea comes from quantitative studies of cerebral architecture. Oliveira et al. reported a decrease in the thickness of normal appearing cortex in patients with localized PMG [33]. Analogously, relatively widespread abnormalities in cortical curvature have been reported in patients with PMG, including within lobes that are not involved by frank dysplasia [34]. Widespread alterations in association pathways in polymicrogyric brains could account for these observations, especially in light of evidence for the importance of corticocortical connections in the development of normal gyral/sulcal morphology [35].

It is important to consider that the output of quantitative functional imaging studies is always closely related to the specific technical parameters utilized during image acquisition, processing, and analysis. For this reason, it is crucial to tie any such imaging findings to an identifiable functional phenotype. We report an association between absence of the left AF and language impairment in a cohort of pediatric patients with PMG. In our study population, all PMG patients with absence of the left AF were language impaired while all subjects with normal language function (both in the PMG and normative groups) had an identifiable left AF. In addition to corroborating the significance of the observed relationship between the locale of dysplastic cortex and intrahemispheric association pathways, these findings suggest that absence of the left AF may be a specific marker of language impairment in this patient population. They further raise the possibility that successful formation of corticocortical connections may be more closely associated with network function than simply whether the relevant cortex is dysplastic. In other words, dysplastic cortex that can support the formation and preservation of intrahemispheric association pathways may also be capable of supporting normal language development. Interestingly, Loui et al. recently demonstrated a failure to identify a superior component of the right AF in 9 of 10 subjects with tone deafness (this component of the right AF was identifiable in all control subjects) [36]. This work suggests that a connectivity-based assessment of network function could potentially be generalized beyond the language network, a possibility which will require future study.

Although our findings suggest that formation of the left AF may be an important step toward the development of normal language function, the observed language impairment in some patients with a left AF suggests that it is not sufficient. This finding is expected given our study design, which evaluated only the relevance of WA and BA. Although these two areas are clearly important, the fluent comprehension and production of language are predicated on the rapid transmission of information among numerous, distributed cortical areas [37]. Furthermore, the AF is not the only white matter pathway connecting the inferior frontal and superior temporal lobes [38]. It is likely that these diverse components subserve a breadth of functional domains that contribute to normal human language function* in toto*. Although insufficient to define the functional relevance of all parts of the network, this study design was adopted in the service of simplicity and the well-defined relationship between two key language cortices and the AF. Future studies correlating individual subcomponents of the network with detailed neuropsychological analyses of a range of language subdomains would be of great value toward extending the relationships suggested by this study.

Although to our knowledge this is the first report on the functional significance of absence of the left AF in patients with MCDs, the importance of the left AF to language function has been suggested in other neurodevelopmental disorders. Abnormal, though identifiable, arcuate fasciculi have been reported in a range of disorders manifesting speech delay [20, 21, 39]. Wilson et al. reported absence of the left AF in six of seven patients with Angelman syndrome, a developmental disorder characterized by pervasive developmental delay and failure to develop speech [40]. Sundaram et al. reported absence of the left AF in 11 of 20 patients with global developmental delay [19]. Both studies reported presence of the left AF in all normal control subjects. These results are in line with ours and suggest that absence of the left AF may be a marker of language impairment in other patient populations. Of note, a study by Lebel and Beaulieu reported a small frequency of absence of the left AF in normal patients [18]. In our normative population, by contrast, all subjects had an identifiable left AF. This discrepancy could be accounted for by the relatively small number of subjects in our study. Alternatively, it could reflect differences in image acquisition (including field strength and directional scheme for diffusion weighting) or methods of image processing. Further studies designed to rigorously define the normal character of the AF at 3 Tesla will be a necessary adjunct to future investigation.

This study has several limitations. First, this is a study of a highly selected cohort of patients with PMG. Extrapolation of these results to patients with other malformations or to other patient groups at risk for language impairment may not be valid. Second, as fMRI was not performed, the actual locations of receptive and expressive language were not definitively ascertained. In a related matter, the small patient number in this study precluded a statistical accounting for handedness. There is, however, some evidence to suggest that lateralization of the AF may not be consistently related to either functional lateralization or handedness [41]. Furthermore, this limitation is substantially mitigated by corroboration with language function. In particular, if there was a significant frequency of ectopic or right hemispheric dominant language function, it did not seem in this study to impact the prognostic significance of the imaging findings. Third, functional assessment of language in this study was limited to a gross three-point scale, chosen to reflect clinically relevant differences in language function while maximizing reproducibility of the assessment. Detailed neuropsychological evaluation was not performed but would be of great potential value to future studies. In particular, such an evaluation might allow the identification of specific domains of language dysfunction in each patient which could further elucidate functional subspecialization within the language network.

## 5. Conclusion

In conclusion, we have used the language network to examine the relationship between the locale of dysplastic cortex and intrahemispheric association pathways in pediatric patients with PMG. In particular, we report the following: (1) an association between the presence of dysplastic cortex within the expected anatomic locations of WA and/or BA and absence of the left AF; (2) a strong association between absence of the left AF and language impairment. These findings suggest that the ability to form and/or maintain corticocortical connections is frequently impaired in polymicrogyria and, furthermore, may be closely related to network function. If these findings can be validated in larger studies, such approaches may form the basis for useful biomarkers of cortical function in patients with malformations of cortical development.

## Figures and Tables

**Table 1:** Diagnostic performance of absence of the left arcuate fasciculus for prediction of language impairment.

	Diagnostic performance	95% LCI	95% UCI
Sensitivity (%)	78	40.1	96.1
Specificity (%)	100	56.1	100
PPV (%)	100	56.1	100
NPV (%)	78	40.1	96.1

LCI: lower limit of the 95 percent confidence interval; UCI: upper limit of the 95 percent confidence interval; PPV: positive predictive value; NPV: negative predictive value.
